# Bringing the examination to the resident: nurse-facilitated teledentistry in Estonian care homes

**DOI:** 10.3389/fdmed.2026.2014381

**Published:** 2026-10-29

**Authors:** Ave Voit, Krista Vapper

**Affiliations:** 1 Estonian Dental Association, Tallinn, Estonia; 2 Heidelberg Institute of Global Health, Section for Oral Health, Heidelberg University Hospital, Heidelberg University, Heidelberg, Germany; 3 West Tallinn Central Hospital, Tallinn, Estonia

**Keywords:** health workforce, long-term care, nurse training, oral health, skill mix, task sharing, teledentistry

## Abstract

Care-home residents have considerable oral health needs yet face some of the greatest barriers to visiting a dental practice. Using Estonia as a case study, this Perspective examines what a health system needs for nurse-facilitated remote oral examination to become routine. In Estonia, a trained care-home nurse films the resident's mouth with an intraoral camera under the guidance of a hospital dentist by live video and enters the findings into the electronic health record. National claims data from January 2024 to July 2026 show that two public hospitals performed 550 teledentistry examinations for 537 residents. Because residents did not need to be transported to a dental clinic, each examination saved an estimated mean round trip of 132 km, equivalent to 2.5–5.4 hours of care-staff time and 22–34 kg of carbon dioxide equivalent. Tariff parity with in-person care, public provision and record integration enabled routine operation but did not create demand: activity remained around five examinations a month until one care-home operator requested examinations across all its homes. Scaling the model in Estonia therefore depends on policy rather than technology. We propose linking existing oral health assessment guidance for care homes to the teleconsultation pathway, informing care-home managers about the service, allowing trained care workers as well as nurses to facilitate examinations, and expanding provider capacity. Remote examination can also guide mobile dental units to care homes with documented unmet treatment need.

## 1 Introduction

Residents of care homes have high and rising oral health needs and are among the least able to access a dental practice (1, 2). A single visit can mean a long round trip in an adapted vehicle, with one or two care workers withdrawn from the home for most of the day. This is a workforce cost for health systems globally that digital models of care can substantially reduce. Drawing on Estonia's recent experience with teledentistry, this Perspective examines how oral health care can reach residents where they live.

The Estonian model maps directly onto global policy. The World Health Organization (WHO) Bangkok Declaration commits Member States to expanding health workers’ capacity for community-based care through task sharing and skill mix, strengthening oral health information systems, and reducing the carbon footprint of oral health care (3). The WHO Global Oral Health Action Plan 2023–2030 names the health workforce and oral health information systems among its strategic objectives (4).

The feasibility of remote oral examination in care homes is already established. Studies from France, the United States and Australia have shown that trained staff can capture intraoral images for a remote dentist, with high diagnostic accuracy against direct examination (5–10). Yet most services have remained time-limited projects outside everyday care (11, 12). The practical question is therefore what a health system must have in place for remote examination to happen routinely rather than occasionally.

Estonia has run a national health information system since 2008 that connects all providers and centralizes patient records. Dental records are part of the system, and the dental software in national use is centrally integrated (13–15). This places Estonia ahead of most countries, where dentistry has been slower than medicine to join national electronic health record frameworks, risking leaving dental data isolated from the wider health system (16). Infrastructure is a prerequisite, but organization and financing determine whether a service is used. This Perspective describes how Estonia organizes and pays for nurse-facilitated examinations, uses national claims data to trace uptake, estimates the care-staff time and travel avoided, and draw outs lessons for workforce policy.

## 2 The Estonian teledentistry service model

The Estonian Health Insurance Fund (EHIF) reimburses teledentistry examinations for care-home residents when a dentist provides a video consultation to a patient within the general care-home service (17). Two large public hospitals provide the examinations: Tartu University Hospital in the south and West Tallinn Central Hospital in the north of Estonia. Provision is deliberately restricted to these two providers to keep the service publicly run and to ensure consistent quality. The model also allows the treatment needs of each resident to be mapped systematically as a basis for planning further care.

Each examination is conducted jointly by a nurse at the care home and a dentist at the hospital, connected by live video call. In the care home, the nurse positions the resident and scans the oral cavity with an intraoral camera while the dentist guides the examination over the video bridge and records the oral health status directly in the dental software, from where it transfers automatically to the patient's health record. Consent follows the ordinary rules for Estonian health services: the resident consents where able, otherwise the legal representative decides.

The EHIF reimburses both the nurse's contribution as an oral cavity assessment by video consultation (€34.65) and the dentist's as a video consultation (€46.90) (Supplementary Material S1, Table S1). The dentist's fee is identical to the in-person fee, so remote delivery carries no financial penalty. A central control mechanism ties the pair together: the nurse's claim can be submitted only after the dentist's claim for the same consultation has been entered.

The EHIF also has tariffs for mobile dental care (Supplementary Table S1). Three mobile units operate in Estonia, run by one municipal and two private clinics under the EHIF contract, and the mobile tariffs cover only screening and prevention for insured children under 19 years of age (18). The quarterly readiness payment compensates a provider for keeping a unit on the road and is paid only if the unit has served at least eight municipalities outside the provider's home county in that quarter (18). No tariff yet covers mobile dental care for adults in care homes.

## 3 The model in practice: uptake and co-benefits

This section draws on publicly available EHIF statistics on reimbursed services under code 52469, covering all reimbursed consultations in Estonia from January 2024 to July 2026, broken down by provider, patient age group and county of residence. The analysis is descriptive and intended only to illustrate the argument of this Perspective article. The avoided travel, staff time and emissions reported below are scenario estimates (see Supplementary Materials S1, S2). Because only aggregated public data were used, ethical review and consent were not required.

### 3.1 Uptake and geography

Over the period the EHIF reimbursed 550 remote dental consultations for 537 people, delivered by West Tallinn Central Hospital (495 consultations, 90%) and Tartu University Hospital (55, 10%), at a dentist-side cost of €25,725. Adding the nurse's paired claim brings the estimated total program cost to approximately €44,900, or €81.61 per examination (Supplementary Material S1). The median patient was 70 years old (range 20–98); 59% of consultations were for patients aged 65 or over and 25% for those aged 85 or over. Consultations for adults under 65 chiefly reflect younger residents of general and special-care homes living with disabling neurological or psychiatric conditions, who depend on the same nurse-facilitated route to care.

Annual volumes were relatively low for almost two years and then increased from 59 consultations in 2024 to 424 in the first seven months of 2026. The increase was demand-driven, because a large care-home management company asked the West Tallinn Central Hospital to conduct oral health examinations of its residents. The hospital delivered 136 teledentistry consultations in April 2026, 55 in May, 67 in June and 135 in July, against a prior baseline of five a month nationally. These four months account for 393 of the 550 consultations (71%) and reached counties across the country, with 101 consultations in Ida-Virumaa, 47 in Lääne-Virumaa and 40 in Valgamaa.

Figure 1 shows, for each county, consultations per 1000 residents aged 65 and over against the round-trip road distance from the county administrative center to the nearer of the two hospitals (19, 20). Nationally there were 1.11 consultations per 1000 older residents over the full period, and 0.27 excluding the four service surge months. Valgamaa (3.55) and Ida-Virumaa (2.34) reached roughly three and two times the national rate. Harjumaa, home of West Tallinn Central Hospital, recorded 0.96. Four counties recorded fewer than 0.3, and Hiiumaa recorded no consultations for residents aged 65 or over. Reach therefore followed care-home demand: one operator's request produced most of the national activity, and counties without such requests stayed near the baseline whether they were near a hospital or far from it (Supplementary Materials S3.1, S3.2).

**Figure 1:**
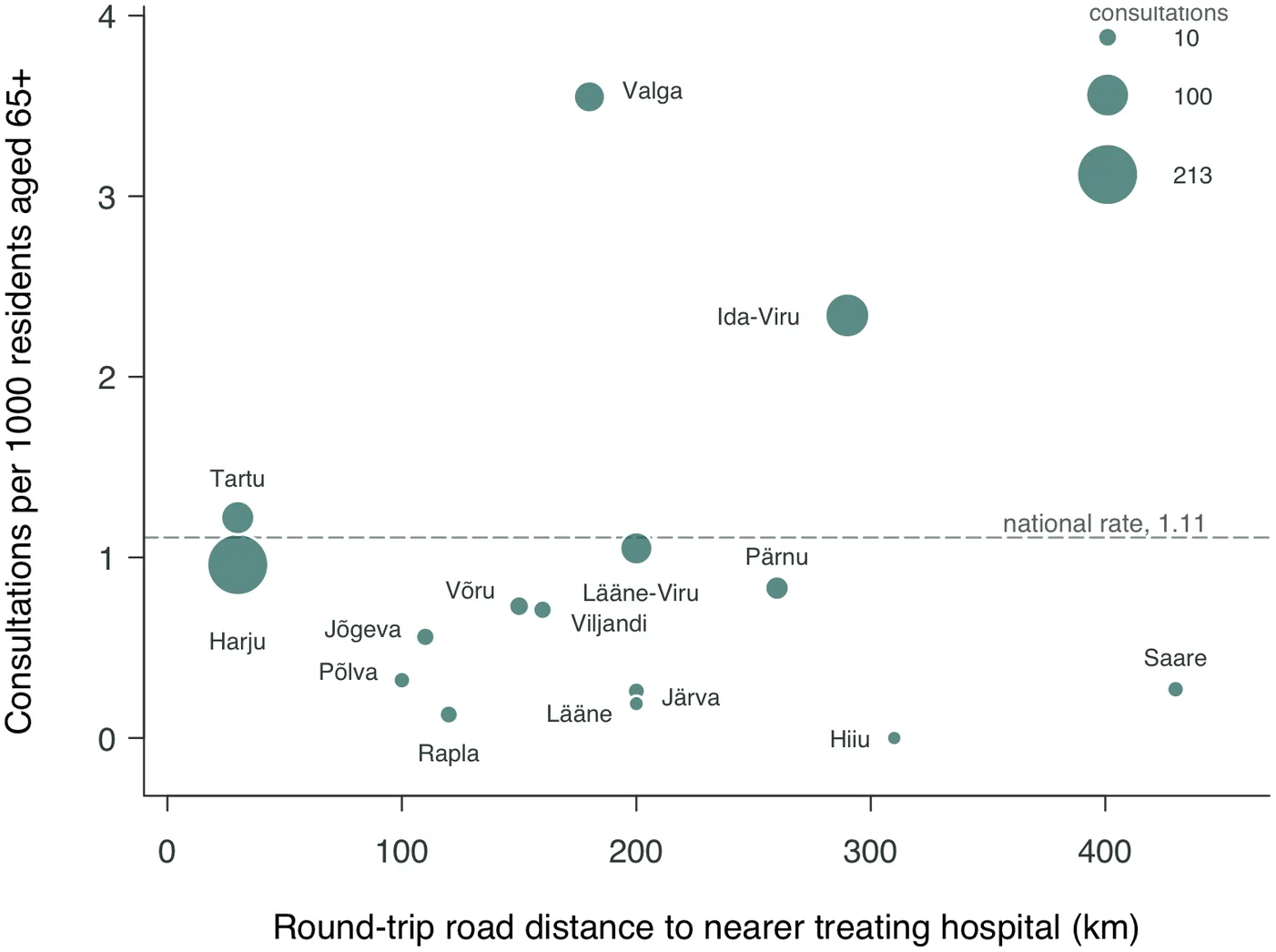
Coverage of the dentist's video consultation tariff (code 52469) by county against round-trip road distance to the nearer treating hospital, January 2024–July 2026. Marker area is proportional to total consultations per county; coverage is expressed per 1000 residents aged 65 and over.

### 3.2 Staff time and emissions avoided

For a care home, the most immediate benefit of remote examination is staff time. Dependent residents are rarely accepted by general dental practices, and those who do reach in-person care commonly need hospital settings, including treatment under general anesthesia (2, 21). The realistic alternative to a remote examination is therefore an escorted journey to hospital dental services. The reference scenario assumes that each consultation displaced a round trip to the nearer of the two providing hospitals, measured by road distance from the county center (19). Within-county journeys were assigned a nominal 30 km round trip, and island-county ferry legs were counted as road distance only. Journeys to the hospital that actually delivered the examination, often the more distant one, form an alternative scenario (Supplementary Material S2).

On this basis the 550 consultations correspond to a mean round trip of about 132 km and a total of up to about 73,000 km. Assuming one carer accompanies each resident for the full round trip and waits about an hour on site, the avoided journeys represent roughly 1,400–3,000 hours of care-staff time, or 0.8–1.7 full-time-equivalent years returned to the homes: 2.5–5.4 hours per examination. Travel time, unlike supervision, is shared when residents travel together. With two carers escorting four residents per vehicle the saving falls to about 600 hours (Supplementary Material S2). Either way, the time returns to the home and its other residents, where the cost of an escorted hospital visit falls.

The same journeys also carry an environmental cost. Applying standard conversion factors (22), the avoided travel corresponds to roughly 12 tonnes of carbon dioxide equivalent (tCO2e) by car and 18 tCO2e by adapted van, or 22–34 kg CO2e per consultation, within the range reported for telemedicine generally (23). Under the actual treating-hospital scenario the total rises to approximately 110,000 km and 18–28 tCO2e, while conservative assumptions lower it to 1.5-2.3 tCO2e (Supplementary Material S2.1).

## 4 Discussion

### 4.1 What Estonia adds to the international experience

Estonia is not the first country to examine care-home residents remotely — what sets it apart is how the service is embedded into the national health system. France's e-DENT service has provided oral teleconsultations from Montpellier University Hospital since 2014, using an asynchronous store-and-forward model, and has reached more than 2,000 people in care homes, prisons and chronic disease services (5, 24). Its national roll-out, however, has been held back by the absence of health insurance remuneration for dentists (12). Estonia started from the opposite position, paying dentists the same tariff as for in-person care. The contrast suggests that reimbursement, more than the clinical model, determines whether teledentistry stays a single-center service or becomes a national one. In the United States, the store-and-forward Virtual Dental Home is reimbursed by California's Medicaid program (7), yet published long-term care implementations remain few, small and hygienist-delivered (8). Australian studies come closest to the Estonian design: care-home nurses and staff took intraoral images under real-time guidance from a dentist, which reduced the need for off-site visits (9, 10). More broadly, a rapid review of teledentistry for homebound and institutionalized older adults found interventions with a mean duration under seven months and none integrated into primary health care (11). A nineteen-country comparison concluded that reimbursement and integration, not technology, decide whether teledentistry reaches practice (25). Estonia's contribution is therefore not the technology but the conditions around it: a tariff equal to in-person care, delivery through public hospitals, and automatic transfer of findings to the national health record. Together, these make the service routine rather than a time-limited project.

### 4.2 Three workforce constraints

Estonia's current teledentistry model faces three constraints, all of them policy choices rather than technical limitations.

Firstly, the teledentistry service uptake largely depends on individual initiative. Our analysis showed that awareness of the service is low, and it scaled only when one care home operator requested it across all of its institutions. The Estonian Social Welfare Act requires care homes to support residents’ daily living, including personal hygiene, but does not mention oral examination (26). At the same time the EHIF has issued a guidance document for care-home staff recommending an oral health assessment by a trained worker on admission, annually, and more often for residents at high risk, with referral to a dentist's consultation when problems are found (27). However, this guidance is not binding and does not link the assessment to the teledentistry service. The essential next step should be to add teleconsultation to the referral pathway and inform care home managers that the service exists, is reimbursed, and requires only one trained member of their own staff.

Secondly, the current model needs a trained nurse to operate the intraoral camera. The EHIF currently contracts nursing care in around 200 general and 58 special-care homes (28), so many but not all homes have the staffing the model requires. International experience shows that a broader skill-mix is feasible for this type of service. Australian programs have trained registered nurses and other care-home staff to capture intraoral images under real-time guidance (9, 10), United States programs train facility staff as well as nurses to operate the camera (29), and a UK project is testing smartphone image capture by care-home staff supported by a dental nurse (30). Estonian guidance already asks trained care-home workers to assess oral health (27). Allowing the same workers to facilitate the examination would extend coverage to homes without a nurse.

Third, the EHIF currently contracts only two hospitals to provide the service for the whole of Estonia. This sufficed at five consultations a month and absorbed a surge of more than a hundred, but it leaves little capacity for further growth. If demand grows as intended, provider capacity must grow with it, either through contracting more providers or allocating more dentist time at the existing two.

### 4.3 From examination to treatment

Remote examination identifies need but does not treat it. Mobile dental care already exists in Estonia, but for a different population: the three mobile units deliver screening and prevention to children under 19, and no tariff covers mobile treatment of adults in care homes (18).

West Tallinn Central Hospital plans from 2027 to take a mobile unit to care homes where remote examinations recorded unmet treatment need. Mobile dental care for care-home residents is well established elsewhere. Belgium's Gerodent program has sent a mobile dental team into homes since 2010, with reduced accumulated treatment need (31). Swiss mobile care ranges from portable equipment to a van-based clinic set up inside the care home (21), at set-up costs around CHF 505,000 (32). In Sweden dental personnel screen residents where they live before mobile teams treat at the bedside (33, 34). In each of these models, treatment need is identified in person. What Estonia would add is targeting: deployment planned from remotely collected data rather than by assumption. Remote examination then becomes the triage layer of a two-step pathway in which need is examined remotely, mapped home by home, and then the clinic travels to the residents rather than the residents to the clinic. The avoided-travel logic extends into treatment: instead of care home staff escorting residents one by one to dental clinics, a mobile unit staffed by a dentist and a dental assistant can treat residents in several homes per day.

### 4.4 Limitations

Innovative health services are often harder to sustain than to launch, and several features of the Estonian service are fragile. Provision rests on two hospitals and in practice largely on one, 71% of activity came from a single operator's request, and facilitation depends on nursing contracts that not all homes have. The tariffs are set by regulation and can be revised, and two and a half years is a short observation period. The claim that can be made is narrower than success: the conditions were sufficient for the service to persist at low volume without project funding and to absorb a twentyfold rise in demand when it came. Whether it becomes the routine first step in care-home dental care depends on the workforce decisions above and on funding for the subsequent treatment pathway.

The analysis behind this Perspective is deliberately lightweight, and several limitations follow. The data show that an examination took place and where the patient lived, but not what it found or what followed. Obtaining aggregate clinical findings from the national health information system is the first priority for further work. The analysis is descriptive, with no comparison group, and a short observation period. The clinical value of the pathway also remains to be established: oral health outcomes, such as untreated caries, pain, tooth loss, oral health-related quality of life and hospital admissions for dental infection, should be investigated alongside the experience of residents and care staff. Finally, the staff-time and emissions estimates are indicative. Distances run between county centers and the hospital cities rather than from individual care homes, ferry crossings are counted as road distance only, the conversion factors are UK averages, and only direct vehicle emissions are counted, excluding the service's own footprint and follow-up journeys for treatment. Future analyses could use care-home-level journey data, Estonia-specific emission factors and a life-cycle approach. The mobile unit rollout would be the first opportunity to test the avoided-travel counterfactual against observed treatment.

## 5 Conclusion

The Estonian experience suggests that remote oral examination of care home residents can move from pilot project to routine service when three conditions are in place: staff reimbursement is at parity with in-person care, provision is public, and findings go automatically to the national health record. For systems that already have the infrastructure, the remaining work is organizational and concerns the workforce: define who in the home may facilitate and train them, make sure managers know the service exists and can ask for it, resource providers to respond, and use the data that remote examination generates to send treatment where it is needed.

## Data Availability

The original contributions presented in the study are included in the article/Supplementary Material, further inquiries can be directed to the corresponding author.
