# Supplementary material for: Bringing the examination to the resident: nurse-facilitated teledentistry in Estonian care homes
Source: Front Dent Med. 2026 Oct 29;7:2014381. doi: 10.3389/fdmed.2026.2014381 (PMC13754676; doi:10.3389/fdmed.2026.2014381)
Supplement: Supplementary file 1 [file Datasheet1.pdf]

Supplementary material

S1.1 Aim, scope and outputs

This section documents how the estimates in Section 3 of the main text were produced. In keeping with the Perspective format of the main text, the analysis is descriptive and retrospective, uses aggregate public data only, and is an illustrative scenario estimate, not a life-cycle assessment or a hypothesis test. It values the 550 remote consultations delivered between January 2024 and July 2026 by the patient journeys they may have replaced. It excludes the emissions of the video consultation itself (devices, network, data center) and the manufacture and maintenance of vehicles.

S1.2 Data

Source	Parameters
EHIF public statistics export, service code 52469 [S1]	Consultations, provider, patient age and county, reimbursed amount
EHIF list of health services (regulation) and tariff history	Tariffs for codes 52469 and 3191
Statistics Estonia, table RV0240 [S2]	Population aged 65+ by county, 1 January 2026
Estonian Transport Administration, national road register [S3]	Road lengths between cities for calculating distances between counties and hospitals
DESNZ greenhouse gas conversion factors 2025 [S4]	Emission factors

Source: Regulation establishing the EHIF list of health services (main text reference 17).

Public statistics export and population. The analysis uses publicly available aggregate statistics from the EHIF for code 52469 (dentist's video consultation), covering every reimbursed instance from January 2024 to July 2026 with no sampling or cohort restriction [S1]. The regulation restricts the code to video consultations by a hospital dentist for a patient receiving nursing care within the general care-home service, so each claim corresponds to one consultation of this service. The record contains 550 consultations for 537 people: 495 (90%) delivered by West Tallinn Central Hospital (WTCH) and 55 (10%) by Tartu University Hospital (TUH). Age and county of residence are taken from the export dataset.

Surge period. 393 of the 550 consultations (71%) fall in April–July 2026 (136, 55, 67 and 135 per month), following a request from one care-home operator to WTCH**.** The remaining 157 consultations (59 in 2024, 67 in 2025, 31 in January–March 2026) form the "excluding surge" sensitivity case.

Nurse-side claims. Nurse-side claims (code 3191) were not used as a data source. A separate EHIF export for the same period contained 546 claims for 528 people (€18,996), which does not reconcile with the 550 dentist-side claims for 537 people, and the two extracts could not be matched at claim or person level [S1]. Nurse-side cost is therefore estimated from the dentist-side record (S1.4).

Table S1. Estonian Health Insurance Fund tariffs for remote and mobile dental care, in force from 1 April 2026.

Code	Service	Price (€)	Code introduced
52400	Dentist's consultation, in person	46.90	—
52469	Dentist's video consultation	46.90	April 2022
3191	Oral cavity assessment within the nursing service, by video consultation	34.65	January 2023
52470	Mobile dental prevention service, dentist [a]	57.15	January 2024
52471	Mobile dental prevention service, nurse [a]	40.00	January 2024
2330K	Mobile dental service readiness payment, per quarter [b]	8,873.12	—

Source: Regulation establishing the EHIF list of health services (main text reference 17).

[a] *Mobile tariffs (codes 52470 and 52471) are currently limited to screening and prevention for insured persons under 19 years; three mobile units operate, run by one municipal and two private clinics under EHIF contract.*

[b] *Compensates a provider for keeping a unit on the road and is paid at most once per quarter to a provider that has delivered the mobile service in at least eight different municipalities outside the county of its main place of business (EHIF Decision No. 416, Annex 10c; main text reference 18). No tariff currently covers mobile care for adults in care homes. The quarterly price history for codes 52469 and 3191 over the observation period follows.*

Tariff history (EHIF list of health services). Used to recover service counts from reimbursed cost (code 52469) and to estimate the nurse-side cost (code 3191).

Period	52469 dentist (€)	3191 nurse (€)
2024 Q1	47.27	37.03
2024 Q2–Q4	49.97	38.63
2025 Q1	44.83	33.49
2025 Q2–Q4	45.27	33.75
2026 Q1	45.40	33.76
2026 Q2–	46.90	34.65

S1.3 Scenarios and counterfactual

Dependent care-home residents are rarely accepted by general dental practices, and those who do reach in-person care commonly need hospital settings, including treatment under general anesthesia. The realistic in-person alternative is therefore a journey to one of the two providing hospitals, and the rational destination is the nearer one.

- **Reference scenario.** Each consultation displaces a single-occupancy road round trip from the patient's county administrative center to the nearer (by road) of Tallinn and Tartu. Counterfactual share = 1.0.
- **Actual-treating-hospital scenario.** The trip goes to the hospital that delivered the consultation. WTCH served 90% of consultations, including counties closer to Tartu, so distances are longer. Reported in S2.
- **Sensitivity cases** (S2): counterfactual share 0.5; four residents per vehicle; excluding the surge period; a combined conservative case; a combined maximal case.

Routing rules apply in all scenarios:

- Within-county journeys in Harjumaa and Tartumaa are assigned a nominal 30 km round trip.
- Island counties (Saaremaa, Hiiumaa) include ferry crossings counted as road distance only. Ferry-specific emissions and fixed time costs are excluded.
- A counterfactual share of 1.0 assumes every consultation displaced a journey. Because some examinations would have been deferred rather than made in person, totals are reported as "up to".

S1.4 Calculations

Avoided distance. For consultation i in county $c(i)$, $D_i = 2 \times$ road distance from the county administrative center to the destination hospital city (30 km for within-county). Total avoided distance $D = \Sigma D_i$.

Emissions. $E = D \times s \times EF \div o$, where s is the counterfactual share, o is vehicle occupancy (residents per trip) and EF is the emission factor. Two factors bound the estimate and are always reported together (S2):

- Car: 0.16725 kg CO2e/km (average car, unknown fuel)
- Van: 0.25430 kg CO2e/km (freight van up to 3.5 t, unknown fuel; proxy for an adapted patient-transport vehicle)

Both are DESNZ 2025 direct (tailpipe) factors, excluding well-to-tank. Per-consultation emissions = E ÷ N, with N = 157 in the excluding-surge case.

Escort time. H = *e* × (V ÷ *v* + *a* × T) − *f* × N, where:

- V = vehicle-km = D ÷ *o*
- T = trips = N ÷ *o*
- *v* = mean speed
- *a* = on-site attendance per trip
- *e* = escorts per trip
- *f* = nurse facilitation time per consultation

FTE-years = H ÷ 1,720. Hours per examination = H ÷ N. Escorts (care home staff) accompany the full round trip and wait on site. The shared-transport case assumes two escorts per four-resident trip, which is 0.5 escorts per resident for both travel and on-site time. The deduction *f* removes nurse time spent facilitating the remote examination, so reported escort hours are net.

Program cost

Dentist-side reimbursement was €25,725.26 (550 claims). Nurse-side cost was estimated by applying the code 3191 tariff in force on each consultation date to the 550 dentist-side consultations, giving €19,163. This is an upper estimate: the control mechanism permits a nurse claim only after the matching dentist claim but does not guarantee one is made. Total program cost is approximately €44,900 (€44,888), or €81.61 per examination, at the tariffs in force on each consultation date. The unreconciled nurse export (€18,996) is 0.9% below the estimate, consistent with it being an upper bound.

Coverage rate. Rate = consultations for patients aged 65+ in county ÷ county population aged 65+ (1 January 2026) × 1,000. It is cumulative over the 31-month period, not annualized, and uses a single population snapshot. National rate: 322 consultations → 1.11 per 1,000; excluding the surge, 80 consultations → 0.27. Marker area in Figure 1 uses consultations at all ages.

S1.5 Parameters

Parameter	Reference	Varied to	Type	Source / rationale
Consultations (N)	550	157 (excl. Apr–Jul 2026)	Data	EHIF claims, code 52469
Destination	Nearer hospital	Actual treating hospital	Assumption	S1.3
Total round-trip distance	72,620 km	110,060 km	Derived	Road distance, county center → hospital city (S3)
Emission factor	0.16725 (car) to 0.25430 (van) kg CO2e/km	Reported as a pair	External	DESNZ 2025 [S4]

Parameter	Reference	Varied to	Type	Source / rationale
Counterfactual share	1.0	0.5	Assumption	Share of consultations otherwise held in person
Vehicle occupancy	1 resident/trip	4 residents/trip	Assumption	A care-home operator may transport several residents together
Escorts per trip	1–2	2 per 4-resident trip	Assumption	Full round trip
On-site attendance	1.0 h per trip	—	Assumption	Escort waits at clinic
Mean travel speed	70 km/h	—	Assumption	Escort-time calculation only
Facilitation time	0.4 h per consultation	—	Assumption	Nurse time facilitating the examination, deducted from the saving
Annual FTE	1,720 h	—	Assumption	Converts hours to FTE-years

S1.6 Validation of distances

Distances are road distances from each county administrative center to the hospital city. They were checked against the national road register, which records the official length of every state road [S3].

S1.7 Exclusions and method limitations

- Origin is the county administrative center, not the care home.
- Emission factors are UK averages, not Estonian.
- Excluded: emissions of the video consultation, vehicle manufacture and maintenance, well-to-tank emissions, ferry-specific emissions and time, and follow-up treatment journeys.
- Counterfactual share of 1.0 is an upper bound.
- The design is descriptive and retrospective, with no comparison group, no individual-level data and no clinical outcomes.

S1.8 Data analysis

All analyses were performed in R 4.6.0 with the readxl package. One script reproduces every reported result from three inputs: the complete EHIF public statistics export for code 52469 (January 2024 – July 2026), the EHIF price list with the tariff history for codes 52469 and 3191, and Statistics Estonia table RV0240. Distances are held in the script as a documented lookup table corroborated against the national road register.

S2. Sensitivity analysis

All scenarios use the calculations in S1.4 and the parameters in S1.5. Emissions are in tCO2e over the full period; per-consultation figures are in kg CO2e. The car and van factors bracket every estimate.

S2.1 Travel and emissions

Scenario	Change from reference	Vehicle-km	tCO2e (car)	tCO2e (van)	kg CO2e per consultation (car–van)
**Reference**	Nearer hospital; 1 resident per trip; counterfactual share 1.0; N = 550	72,620	12.1	18.5	22–34
Actual treating hospital	Destination = hospital that delivered the consultation	110,060	18.4	28.0	33–51
Counterfactual share 0.5	Half of consultations displace a journey	36,310	6.1	9.2	11–17
Occupancy 4	Four residents per vehicle	18,155	3.0	4.6	5.5–8.4
Excluding surge	April–July 2026 removed; N = 157	9,630	1.6	2.4	10–16
**Combined conservative**	Nearer hospital, counterfactual 0.5, occupancy 4; N = 550	9,078	1.5	2.3	2.8–4.2

The actual-treating-hospital scenario is also the maximal case. The reference already assumes counterfactual share 1.0 and single occupancy, so nothing can move it higher. Across all scenarios, total emissions range from 1.5 tCO2e (conservative, car) to 28.0 tCO2e (actual hospital, van). Vehicle occupancy is the largest single lever. It is also the assumption most worth establishing empirically, because shared transport is plausible in exactly the care-home setting this service serves.

S2.2 Escort staff time

Hours follow the formula in S1.4 and are net of 0.4 h nurse facilitation per consultation. FTE-years use 1,720 h. All escort cases use counterfactual share 1.0.

Scenario	Hours	FTE-years	Hours per examination
Reference, 1 escort per resident	1,367	0.8	2.5
Reference, 2 escorts per resident	2,955	1.7	5.4
Shared transport: 2 escorts per 4-resident trip	574	0.3	1.0
Actual treating hospital, 1–2 escorts per resident	1,902–4,025	1.1–2.3	3.5–7.3

Sharing transport cuts gross escort hours (before the facilitation deduction) from 1,587 to 794 relative to one escort per resident, and from 3,175 to 794 relative to two. Unlike vehicle-km, escort time does not fall in proportion to occupancy, because it depends on how many escorts each vehicle carries.

S3. Reach and avoided patient travel by county of residence

Code 52469, January 2024 – July 2026. Counties are ordered by coverage rate. Definitions are in S1.4. WTCH = West Tallinn Central Hospital; TUH = Tartu University Hospital.

S3.1 Reach

County	Consultations (all ages)	of which WTCH / TUH	Aged 65+	Population aged 65+	Per 1,000 aged 65+
Valgamaa	47	44 / 3	25	7,039	3.55
Ida-Virumaa	105	105 / 0	89	37,957	2.34
Tartumaa	55	26 / 29	36	29,410	1.22
Lääne-Virumaa	51	48 / 3	15	14,244	1.05
Harjumaa	213	201 / 12	114	118,705	0.96
Pärnumaa	22	21 / 1	17	20,482	0.83
Võrumaa	12	10 / 2	6	8,254	0.73
Viljandimaa	9	9 / 0	8	11,267	0.71
Jõgevamaa	9	9 / 0	4	7,141	0.56
Põlvamaa	5	2 / 3	2	6,240	0.32
Saaremaa	5	5 / 0	2	7,438	0.27
Järvamaa	6	4 / 2	2	7,657	0.26
Läänemaa	2	2 / 0	1	5,317	0.19
Raplamaa	8	8 / 0	1	7,481	0.13
Hiiumaa	1	1 / 0	0	2,312	0.00
**All Estonia**	**550**	**495 / 55**	**322**	**290,944**	**1.11**

Rates divide the 322 consultations for patients aged 65+ by the county population aged 65+ at 1 January 2026 [S2]. They are cumulative over the observation period, not annualised. Numerator and denominator are shown so rates can be checked. Excluding the surge, the national rate is 0.27 per 1,000 (80 consultations). Rates based on fewer than 10 consultations should be read as counts.

S3.2 Avoided patient travel

County	Round trip to Tallinn (km)	Round trip to Tartu (km)	Nearer hospital	Avoided, reference (km)	Avoided, actual hospital (km)
Valgamaa	490	180	Tartu	8,460	22,100
Ida-Virumaa	320	290	Tartu	30,450	33,600
Tartumaa	370	30	Tartu	1,650	10,490
Lääne-Virumaa	200	240	Tallinn	10,200	10,320
Harjumaa	30	370	Tallinn	6,390	10,470
Pärnumaa	260	350	Tallinn	5,720	5,810

County	Round trip to Tallinn (km)	Round trip to Tartu (km)	Nearer hospital	Avoided, reference (km)	Avoided, actual hospital (km)
Võrumaa	510	150	Tartu	1,800	5,400
Viljandimaa	320	160	Tartu	1,440	2,880
Jõgevamaa	290	110	Tartu	990	2,610
Põlvamaa	490	100	Tartu	500	1,280
Saaremaa	430	"—"	Tallinn	2,150	2,150
Järvamaa	200	240	Tallinn	1,200	1,280
Läänemaa	200	"—"	Tallinn	400	400
Raplamaa	120	"—"	Tallinn	960	960
Hiiumaa	310	"—"	Tallinn	310	310
**All Estonia**				**72,620**	**110,060**

Tartu distances are not shown for counties where Tallinn is the nearer hospital and all consultations were delivered by WTCH, as they enter neither scenario. Mean avoided round trip per consultation is 132 km in the reference scenario and 200 km in the actual-hospital scenario. Distances run from the county administrative centre and follow the routing rules in S1.3 (30 km within-county; ferry legs as road distance only) and the validation in S1.6. The reference column is consultations × the nearer round trip. The actual-hospital column weights each hospital's consultations by its own round trip.

References in the Supplementary Material

S1. Estonian Health Insurance Fund public statistics database. [cited 2026 Sep 16]. Available from: https://tervisekassa.ee/koik-teenused

S2. Statistikaamet [Statistics Estonia]. Table RV0240: Population, 1 January, by sex, place of residence, year and age. [cited 2026 Sep 16]. Available from: https://andmed.stat.ee/et/stat/rahvastik__rahvastikunaitajad-ja-koosseis__rahvaarv-ja-rahvastiku-koosseis/RV0240

S3. Transpordiamet [Estonian Transport Administration]. Teeregister [National road register], export of 16 September 2026. [cited 2026 Sep 16]. Available from: https://teeregister.ee/reet/search

S4. Department for Energy Security and Net Zero. Greenhouse gas reporting: conversion factors 2025. London: DESNZ; 2025 Jun 10. [cited 2026 Sep 16]. Available from: https://www.gov.uk/government/publications/greenhouse-gas-reporting-conversion-factors-2025
